# Radiation and Dust Sensor for Mars Environmental Dynamic Analyzer Onboard M2020 Rover

**DOI:** 10.3390/s22082907

**Published:** 2022-04-10

**Authors:** Victor Apestigue, Alejandro Gonzalo, Juan J. Jiménez, Justin Boland, Mark Lemmon, Jose R. de Mingo, Elisa García-Menendez, Joaquín Rivas, Joaquín Azcue, Laurent Bastide, Nuria Andrés-Santiuste, Javier Martínez-Oter, Miguel González-Guerrero, Alberto Martin-Ortega, Daniel Toledo, Francisco Javier Alvarez-Rios, Felipe Serrano, Boris Martín-Vodopivec, Javier Manzano, Raquel López Heredero, Isaías Carrasco, Sergio Aparicio, Ángel Carretero, Daniel R. MacDonald, Lori B. Moore, María Ángeles Alcacera, Jose A. Fernández-Viguri, Israel Martín, Margarita Yela, Maite Álvarez, Paula Manzano, Jose A. Martín, Juan C. del Hoyo, Manuel Reina, Roser Urqui, Jose A. Rodriguez-Manfredi, Manuel de la Torre Juárez, Christina Hernandez, Elizabeth Cordoba, Robin Leiter, Art Thompson, Soren Madsen, Michael D. Smith, Daniel Viúdez-Moreiras, Alfonso Saiz-Lopez, Agustín Sánchez-Lavega, Laura Gomez-Martín, Germán M. Martínez, Francisco J. Gómez-Elvira, Ignacio Arruego

**Affiliations:** 1 Instituto Nacional de Técnica Aeroespacial INTA, 28850 Torrejon de Ardoz, Spain; gonzaloma@inta.es (A.G.); jimenezmj@inta.es (J.J.J.); mingomj@inta.es (J.R.d.M.); garciamee@inta.es (E.G.-M.); rivasaj@inta.es (J.R.); azcuesj@inta.es (J.A.); andressn@inta.es (N.A.-S.); martinezoj@inta.es (J.M.-O.); amarric@inta.es (A.M.-O.); toledocd@inta.es (D.T.); fjalvarez@eme-es.com (F.J.A.-R.); serranosf@inta.es (F.S.); martinvb@inta.es (B.M.-V.); javier.manzanovazquez@external.thalesaleniaspace.com (J.M.); lopezhr@inta.es (R.L.H.); carrascobi@inta.es (I.C.); carreteroa@inta.es (Á.C.); alcacerama@inta.es (M.Á.A.); fdezvjaharo@gmail.com (J.A.F.-V.); martinbi@inta.es (I.M.); yelam@inta.es (M.Y.); alvarezat@inta.es (M.Á.); martinmja@inta.es (J.A.M.); hoyogjc@inta.es (J.C.d.H.); reinam@inta.es (M.R.); manfredi@cab.inta-csic.es (J.A.R.-M.); viudezmd@cab.inta-csic.es (D.V.-M.); gomezml@inta.es (L.G.-M.); gomezej@inta.es (F.J.G.-E.); arruegori@inta.es (I.A.); 2 Jet Propulsion Laboratory, California Institute of Technology, Pasadena, CA 91109, USA; justin.s.boland@jpl.nasa.gov (J.B.); danmac@jpl.nasa.gov (D.R.M.); lori.moore@jpl.nasa.gov (L.B.M.); manuel.delatorrejuarez@jpl.nasa.gov (M.d.l.T.J.); christina.hernandez@jpl.nasa.gov (C.H.); elizabeth.c.cordoba@jpl.nasa.gov (E.C.); robin.m.leiter@jpl.nasa.gov (R.L.); arthur.d.thompson@jpl.nasa.gov (A.T.); soren.n.madsen@jpl.nasa.gov (S.M.); 3 Space Science Institute, 4765 Walnut St, Suite B, Boulder, CO 80301, USA; mlemmon@spacescience.org; 4 Ingeniería de Sistemas para la Defensa de España (ISDEFE), Beatriz de Bobadilla St, 3, 28040 Madrid, Spain; bastidel.pers_externo@inta.es (L.B.); cu4570_3.pers_externo@inta.es (M.G.-G.); manzanorp.pers_externo@inta.es (P.M.); urquiomr@cab.inta-csic.es (R.U.); 5 Centro de Astrobiología (INTA-CSIC), 28850 Torrejon de Ardoz, Spain; 6 NASA Goddard Space Flight Center, Greenbelt, MD 20771, USA; michael.d.smith@nasa.gov; 7 Department of Atmospheric Chemistry and Climate, Institute of Physical Chemistry Rocasolano, Consejo Supeior de Investigaciones Científicas (CSIC), 28006 Madrid, Spain; a.saiz@csic.es; 8 Departamento Física Aplicada I, Escuela Superior de Ingenieros, Universidad del País Vasco, Alameda Urquijo St, 48013 Bilbao, Spain; agustin.sanchez@ehu.es; 9 Lunar and Planetary Institute, Universities Space Research Association, Houston, TX 77058, USA; gmartinez@lpi.usra.edu

**Keywords:** Mars, Mars 2020, MEDA, RDS, instrumentation, atmosphere, dust, clouds, ozone

## Abstract

The Radiation and Dust Sensor is one of six sensors of the Mars Environmental Dynamics Analyzer onboard the Perseverance rover from the Mars 2020 NASA mission. Its primary goal is to characterize the airbone dust in the Mars atmosphere, inferring its concentration, shape and optical properties. Thanks to its geometry, the sensor will be capable of studying dust-lifting processes with a high temporal resolution and high spatial coverage. Thanks to its multiwavelength design, it will characterize the solar spectrum from Mars’ surface. The present work describes the sensor design from the scientific and technical requirements, the qualification processes to demonstrate its endurance on Mars’ surface, the calibration activities to demonstrate its performance, and its validation campaign in a representative Mars analog. As a result of this process, we obtained a very compact sensor, fully digital, with a mass below 1 kg and exceptional power consumption and data budget features.

## 1. Introduction

The Mars 2020 mission [1] meets the long-term science goals of the NASA Mars Exploration Program. These goals are (i) to determine whether life ever arose on Mars or if the environment was ever suitable for sustaining life, (ii) to characterize the present and recent-past climate of the planet and its geology, and (iii) to plan the future robotic missions and the human arrival. Spain contributes to the mission’s scientific payload through the SuperCam Calibration Target [2] and the Mars Environmental Dynamics Analyzer (MEDA) [3], an advanced environmental station whose instruments characterize the red planet’s weather. MEDA station, along with REMS (Rover Environmental Monitoring Station) [4] and TWINS stations (Temperature and Winds for Insight) [5] onboard Curiosity rover and InSight, respectively, provide weather measurements on the surface of Mars at three different locations simultaneously.

MEDA is comprised of several sensors: (i) five atmospheric temperature sensors (ATS); (ii) a thermal infra-red sensor for ground and sky temperature measurements (TIRS); (iii) a humidity sensor (HS); (iv) a pressure sensor (PS); (v) two wind sensors to measure airspeed and direction; (vi) a solar radiometer radiation and dust sensor (RDS). Furthermore, MEDA includes an instrument control unit (ICU) located in the rover’s bay to control the different sensors’ activation and measurement sequence. This unit also works as the interface with the rover computer, allowing MEDA to operate independently of the rover through the station programmable observation tables (OT).

One of the main MEDA goals is to characterize the suspended dust in the atmosphere by obtaining information on the vertical distribution and optical properties of dust particles. These properties determine the scattering characteristics of airborne dust, affecting the absorption and reflection of the solar radiation and, thus, the energy balance that drives the planet’s atmospheric dynamics. Besides, the dust particles can act as ice nuclei [6] to form clouds, which influences the vertical distribution of dust (along with dynamics) by cloud scavenging. Dust and clouds are both indicators and drivers of atmospheric processes with complex feedback mechanisms, which points out the need to characterize dust and clouds’ properties simultaneously over different periods and locations. Thus, in situ weather observations are essential for our understanding of the planet’s climate [7] and the validation of different atmospheric models from general circulation [8] to local scales [9].

Since the Viking missions era, the concentration and optical properties of Martian dust have been retrieved by instruments onboard landers and rovers. Several cameras included in different missions, dedicated initially to studying the terrain’s geology or to the maneuvering of the rovers, played a key role in investigating airborne dust. These cameras can measure the sky brightness at different angular locations and thus provide constraints on the dust properties at different time periods for a given sol. For instance, Vikings’ panoramic cameras [10] were used to retrieve the aerosol optical depth (AOD) along the day for 1.3 Martian years (the duration of the mission). These results allowed the seasonal variability of the dust cycle to be constrained. Subsequently, AOD measurements were derived from the imager for Mars pathfinder (IMP) at higher temporal resolution (one picture per hour) and at four different wavelengths: 450, 670, 883, and 989 nm [11]. The next landed AOD observations were obtained by the Pancam cameras onboard both Mars Exploration Rovers (MERs). These AOD observations are the most extensive compilation derived from ground observations [12]. They include more than 2200 sols for Spirit and 4300 for Opportunity. In addition to these observations, since 2012, AOD estimations are being carried out by the Mastcam camera onboard the Mars Science Laboratory (MSL) rover [13] and using the navigation cameras [14].

Besides the cameras, airborne dust particles have been studied by two other surface instruments. On the one hand, the miniature thermal emission spectrometers (Mini-TES) onboard MERSs [15] were designed to determine the mineralogy of rocks and soils and characterize the atmosphere’s lower boundary layer. For at least one Martian year and a half (from the end of—Mars year—MY 26 to the middle of MY 28), Mini-TES retrieved 9-µm dust opacity for both rovers traverse areas, as reported in [16,17]. On the other hand, the ultra violet sensor (UVS), integrated into the REMS meteorological station onboard MSL, has been providing the ultraviolet down-welling flux (between 200 to 380 nm) at Mars’ surface since MY 31. This essential astrobiological measurement allows us to retrieve the AOD at different instruments’ wavelengths as reported in [18], or even the particle size and its seasonal and interannual variability [19], in combination with the rover’s mast camera (Mastcam) instrument. 

The common denominator in all previous instruments is that they were not exclusively designed to study the Martian aerosols. Also, their availability and the data budget for those atmospheric studies were, in general, limited, as they were shared between different scientific objectives. However, RDS is the first surface radiometer fully dedicated to studying the Martian atmosphere.

The main scientific objectives of RDS are (a) to estimate the optical and scattering properties of airborne dust as a function of season and local time; (b) to detect dust lifting events near the surface such as dust devils; (c) to detect and characterize clouds, and (d) to estimate the column abundance of ozone as a function of season and local time.

**Figure 1:**
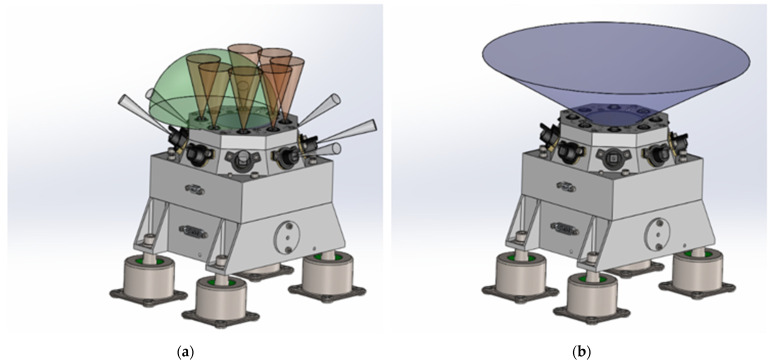
RDS assembly with field of view detail for each detecting technology: (**a**) RDS-discrete photodetectors; (**b**) RDS-SkyCam.

With theses objectives, RDS combines in an integrated device two different but complementary measurement strategies based on two detecting technologies (Figure 1):

- A sky-pointed CCD-based (charge-coupled device) camera (RDS-SkyCam), delivered by the Jet Propulsion Laboratory (JPL), designed to take sky pictures at different times during the day in the 600–800 nm visible to near-infrared range.
- A digital radiometer, RDS-discrete photodetectors (RDS-DP), consisting of two sets of photodiodes: (i) a set of 8 detectors pointed at the zenith (TOP detectors) at different spectral bands from ultraviolet to near-infrared (245, 295, 250–400, 450, 650, 750, 950, and 110–1100 nm), and (ii) a set of 8 detectors pointing sideways (LAT detectors) with an elevation of 20° above the horizon (except LAT8 35°), all of them centered on same wavelength 750 nm and distributed uniformly around the RDS (45° azimuthal spacing). RDS-DP sensors’ configuration was selected to study the optical properties of dust and clouds at different spectral bands and determine the column abundance of ozone for the first time at the Martian surface.

Both technologies coexist in the same sensor, making RDS a powerful tool for characterizing the diurnal dust cycle, providing MEDA the opportunity to operate them independently and/or simultaneously. The camera has a high cost in terms of data budget and power consumption, which reduces its temporal resolution due to the limited resources of memory, radio link, and energy. However, RDS-SkyCam offers high-quality images of the sky, enabling derivation of a complete set of atmospheric parameters per image and providing context for other investigations related to dust devils or clouds. On the other hand, the RDS-DP provides complex information to retrieve, which requires the use of inversion models. Still, it is efficient in terms of power consumption and data generation. This enables photodetectors to monitor the atmosphere all day and may provide information about short-time events like dust devils and saltation processes alone or in combination with other MEDA sensors.

In the following sections, we describe the RDS technical and scientific characteristics. In Section 2, we show the sensor design in depth. The characteristics of the integration and qualification processes of the instrument are detailed in Section 3. In Section 4, we introduce the calibration process and report the final instrument performances. Section 5 is dedicated to describing the retrieval procedures to be used on Mars. Section 6 validates some of the proposed retrievals, reporting the results of a field campaign performed in a Martian analog. Finally, in Section 7, we summarize and offer a conclusion of the presented work.

## 2. Sensor Description

### 2.1. RDS Rover’s Accommodation

The RDS accommodation on the rover was the first step of its complex and iterative design process. The early sensors’ concepts were adapted to meet the evolving accommodation necessities, sometimes for visual interferences of the RDS side-pointed or lateral optical channels, and other times for updates to the environmental requirements.

**Figure 2:**
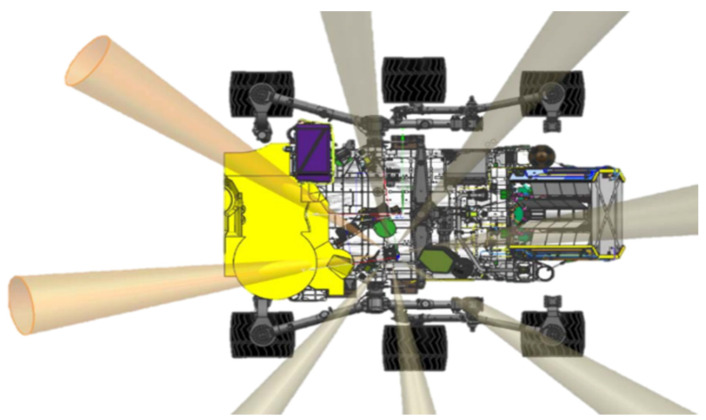
RDS final position on the M2020 rover with its lateral channels FoV represented.

Two lateral photodetector channels had to be modified because of the sample and caching system (SCS) envelope (Figure 2 yellow area). SCS volume entirely covered LAT 1 and part of LAT 8 channel’s FoV. It was decided then to blind the LAT 1 and use its dark signal to estimate the photodetector’s performance degradation due to displacement damage induced by high energy space radiation [20,21]. However, LAT 8 was “saved,” pointing it up to 35° above the horizon, avoiding interferences with the sampling hardware.

**Figure 3:**
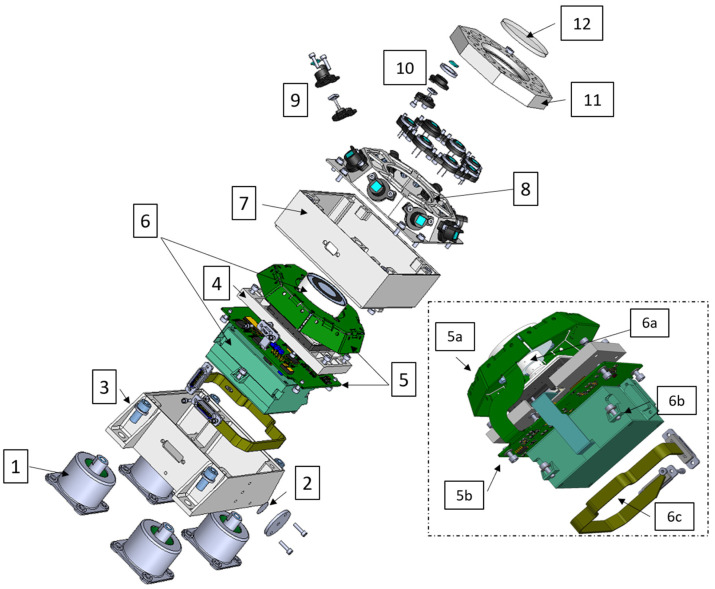
RDS exploded view: (1) shock supressors, (2) HEPA filter, (3) lower-level structure, (4) camera base plate, (5) discrete photodetector PCB, (6) camera subassemblie, (7) intermediate-level structure, (8) upper level structure, (9) lateral optical channel subassemblie, (10) top channel subassemblie, (11) octogonal top cover, and (12) sapphire window. The rectangle includes a detail (reversed view) of the tight integration between RDS-DP and SkyCam sub-assemblies: (5a) semi-rigid RDS-DP optical head PCB, (5b) semi-rigid RDS-DP processing electronics PCB, (6a) Skycam optical head, (6b) Skycam electronic box, and (6c) Skycam I/F internal cable.

On the other hand, during the rover’s design phase, the RDS accommodation area was reconsidered from pyro-shock zone 3 (3000 g) to zone 6 (6000 g) due to the Skycrane slings cutting at landing. At that moment, the complete re-design of the sensor was not a feasible option. It was decided then to include shock suppressors (Figure 3(1)) to reduce the loads to values compatible with the RDS design. The selected isolators are from Barry Controls^®^ T-mount series, which had a previous heritage for similar applications. The version used (T44-AB-10) can cope with a maximum sustained load per isolator of around 45N and is based on an elastomer of hi-damp silicone with an approximated damping factor 0.15. In the elastomeric bulk, a stem with a ¼-28 UNF through holed is embedded to provide an attachment point. The whole assembly is encased within a two-part AISI 302 housing.

The final accommodation location of the RDS was in the middle left of the rover’s deck (see Figure 2). The vertical photodetectors with ±15° FoV have an unrestricted view of the sky in all the possible rover configurations. However, the total light photodetector (TOP 7 with 90° FoV), SkyCam, and some of the lateral channels could be partially covered by movable elements like the high gain antenna or the rover’s articulated mast hardware. In any case, these non-desirable situations represent a small percentage of the total observation time, which will be minimized using “flight-rules” to synchronize these interference positions with moments when MEDA is not observing with RDS or just by having knowledge of that interference.

### 2.2. RDS Mechanical and Thermal Design

The RDS mechanical design provides a compact solution to accommodate both detection technologies and their electronics inside the sensor. RDS-SkyCam is a residual from MSL, so its design was a known input to the RDS design. The SkyCam subassembly consists of two boxes (the electronic box and the optical head) linked with a fixed flexible cable. The rest of the RDS then grows around the camera with a high integration and miniaturization level. With this aim, the main structure has been designed employing several modules in a cubic-shaped case of aluminum 7075. The structure is comprised of three parts, making up the lower, intermediate, and upper levels of the instrument, closed by an octagonal cover on top.

The lower-level structure (Figure 3(3)) houses the camera electronics (SkyCam E-box, Figure 3(6b)) and its electrical harness (Figure 3(6c)). The intermediate-level structure (Figure 3(7)) supports what serves as a base plate (Figure 3(4)) for the RDS-SkyCam optical head (Figure 3(6a)), allowing the alignment of the camera within the required tolerances by shimming at the mounting points. The RDS-DP processing board (Figure 3(5b)) is also screwed to this intermediate structure. The upper-level structure (Figure 3(8)) holds the RDS-DP proximity electronics (Figure 3(5a)) and acts as a frame for the eight lateral photodetector assemblies’ attachments (Figure 3(9)).

Finally, the octagonal top cover (Figure 3(11)) supports the eight top photodetector assemblies (Figure 3(10)). A central opening allows the necessary FoV for the SkyCam. A sapphire bonded by RTV 566 silicone (Figure 3(12)) closes this opening and shields the RDS interiors from the martian dust and the possible impact of pebbles during the landing. 

The thermal design’s primary goal is to maintain the internal RDS components within their required temperature limits at any mission scenario. Additionally, the design is aimed at decoupling the RDS from the rover deck, hence limiting the heat flux exchanged between them.

The small, −55 °C to +50 °C operational range of the RDS-SkyCam electronics led to the inclusion of a heater controlled by MEDA ICU within the RDS-SkyCam electronic box (Figure 3(6b)). This part is in turn insulated from the RDS structure through a stack of titanium washers at the mounting points and highly reflective coating. This allows its operating temperature to be maintained above the required −55 °C while minimizing the heater’s energy required.

The rest of the RDS thermal design relies on passive elements to maintain the internal components’ temperatures within their allowable ranges. Accordingly, the aluminum case’s inner part is finished in low emissivity chromate (Alodine 1200S). The external surfaces are painted white (MAP SG121FD), which reduces the solar heat load absorbed when it allows the rejection of the internal waste heat through IR radiation, thus preventing, for instance, the camera’s electronics from overheating in hot scenarios. 

The bottom face remains chromated to minimize the radiative exchange between the instrument and the rover top deck. Furthermore, the conductive resistance between the RDS and the rover deck dramatically increases when including the elastomeric shock absorbers. 

Contrary to the afformentioned passive insulation strategy, the photodetectors’ FoV masks had to be painted in black (Aeroglaze Z306 paint) to satisfy the optical-performance requirements, as explained in the following section.

### 2.3. RDS Discrete Photodetectors

The RDS-DP design is mainly based on a previous radiometer developed for ExoMars 2016: the Solar Irradiance Sensor (SIS) [22]. This device was included as a part of the DREAMS (Dust Characterization, Risk Assessment, and Environment Analyzer on the Martian Surface) meteorological package [23], the only scientific payload of the Schiaparelli lander. 

The RDS design inherits many SIS features, such as high performance, low power consumption, and miniaturization. However, RDS’ temperature operational range has been extended to be compatible with the mission’s worst cold case scenarios, enabling the sensor to work at −140 °C without using internal heaters. The next subsections describe the key elements of its design.

#### 2.3.1. Optomechanical Design

**Figure 4:**
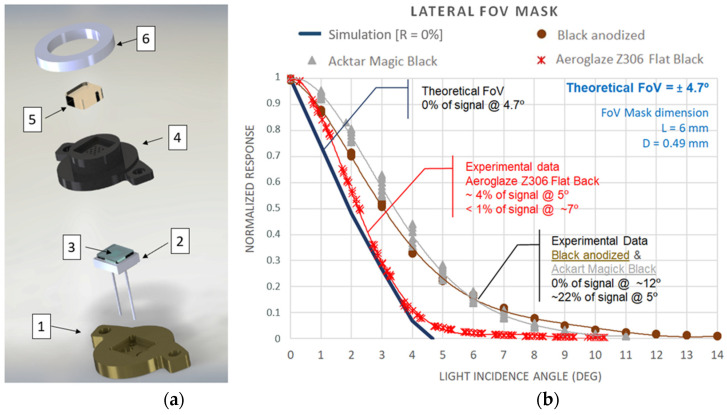
RDS optomechanical sets: (**a**) detail of the exploded view of a TOP channel subassembly: (1) radiation shield, (2) photodiode, (3) interferential filter, (4) FoV mask, (5) sapphire window, and (6) samarium–cobalt magnet; (**b**) normalized signal of a photodetector as a function of the light incidence angle for several FoV masks with the same mechanical dimensions but different surface treatments.

Both top and lateral optical channels use the same approach, maximizing the integration level of the sensor. The optomechanical set’s central element is a silicon photodetector with a 2.4 × 2.4 mm^2^ photosensitive area (Hamamatsu 1337-33bq, Figure 4(a2)) with high sensitivity even in the UV spectrum, providing a spectral response range from 190 to 1200 nm. Its quartz window (except for the TOP 7) has been replaced by an interferential filter (Figure 4(a3)) developed ad hoc for the RDS.

**Figure 5:**
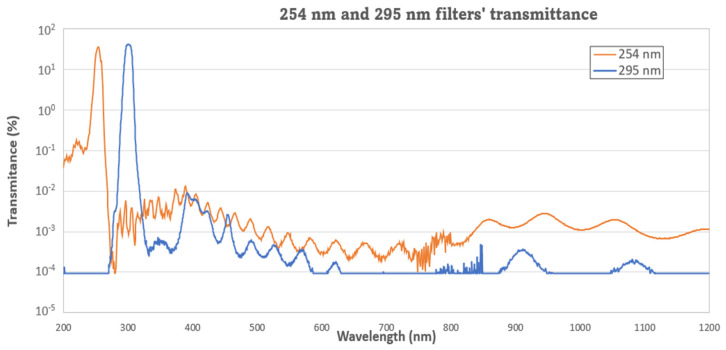
UV interferential filters final performance.

Interference filters have been selected due to their high transmittance in the passband, the large rejection band that avoids the need to stack multiple filters, and their endurance against radiation. They were manufactured by multilayer stacks of different dielectric materials: Nb_2_O_5_, TiO_2_, Ta_2_O_5_, SiO_2_, and HfO_2_. In the case of UV channels (Figure 5), the use of metallic layers (Al) was needed to improve the filter performance. For all of them, the effective refractive index was specified as high enough to minimize the shifting of the filter’s wavelength response due to the angle of light: up to 15 degrees in TOP detectors and 5 degrees for LAT ones restricted by the FoV masks. The effects of transmittance and wavelength shifting due to thermal excursions (120 K) were specified to be less than 0.5% and less than 1 nm, respectively.

The FoV of each set is achieved by a drilled aluminum case (FoV mask set, Figure 4(a4). The relationship between the mask’s thickness and the hole’s diameter determines, theoretically [24], the maximum light angle that reaches the active area of the photodiode. Our interest was to maintain an optical design as compact as possible. Consequently, we tried to minimize the mask thickness in balance with the minimum diameter that can be manufactured by standard drilling. Finally, to improve the signal strength, a drill pattern is applied to the mask to increase photons that impact the silicon area with the same FoV.

In the initial mask’s design, we used black anodize to avoid internal light reflections as it was used in DREAMS-SIS design. This coating offers good behavior at normal incidence light, but its reflectivity increases with the angle showing extended tails on its angular response. This issue is less important for a wide FoV like SIS has. Still, it was crucial for RDS LAT ones where the widening of the mask implies double the FoV, losing azimuthal resolution (Figure 4 shows this effect). The theoretical FoV was compared with the experimental data obtained from identical masks with different surface treatments. Aeroglace Z306 black paint was the coating selected for this application with acceptable reflection effects from a 5° light incidence angle.

Finally, the optomechanical sets have a sapphire window (Figure 4(a5)), preventing dust deposition into the FoV mask’s drills. This strong material will protect the channels during the landing phase, too, against pebbles and little rocks that the Skycrane motors could raise. On the bottom, optomechanical sets end in a case (Figure 4(a1)) that shields the RDS with 1 mm of aluminum thickness, protecting its interior from space radiation. To avoid the deposition of dust over the sapphires, the TOP sets include a strong samarium–cobalt magnet (Figure 4(a6)) as was done for the REMS UVS sensor. Additionally, a passive sunshade protruded in the LAT’s FoV masks is used (Figure 3(9)) to minimize this issue in combination with a high inclination surface.

#### 2.3.2. Electronic Design

**Figure 6:**
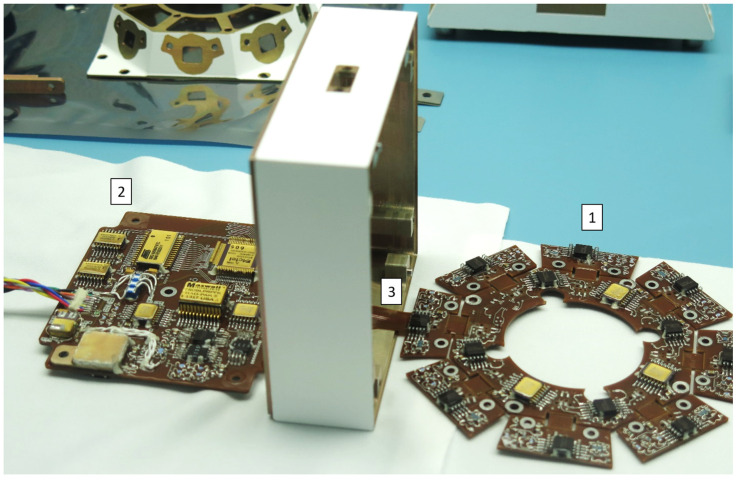
First step of the RDS integration process. It can be seen the RDS-DP optical head (1) PCB, the processing electronics PCB (2) and the flex cable (3) that links both.

As previously mentioned, the fact that the camera was already designed has been a critical design constraint for the RDS-DP side. Photodetectors’ electronics were designed in a rigid-flex PCB (Figure 6). This improves the integration process, containing two main rigid-areas linked with a flexible area (Figure 6(3)): (i) the optical head (OH) and (ii) the processing electronics (PE). The first, the OH (Figure 6(2)) has an octagonal shape with an aperture in its center. Each of the octagon’s sides deploys a small PCB linked by a little portion of flex, enabling the ensemble to fit inside the upper-level structure.

The OH serves as the socket for the 16 photodetectors, and for the 8 temperature sensors (PT1000 class A-type and 2.3 mm × 1.6 mm in size), four of them located in the odd TOP channels and the other four in the even LAT ones. The tight PCB space harbors the proximity electronics for those channels: a trans-impedance scheme for the optical sensors and voltage amplification for the temperature ones. An ultra-low noise amplifier was selected for both (only 0.5 µV peak-to-peak), with low drift (less than 0.005 µV/°C), low offset (below 1 µV), low input bias current (500 pA), and low input offset current (400 pA). The conditioned signals are distributed through three 8:1 multiplexors, and its output and control signals, together with OH’s power and reference lines, are sent across the flexible link to the processing electronics (PE) rigid board.

**Figure 7:**
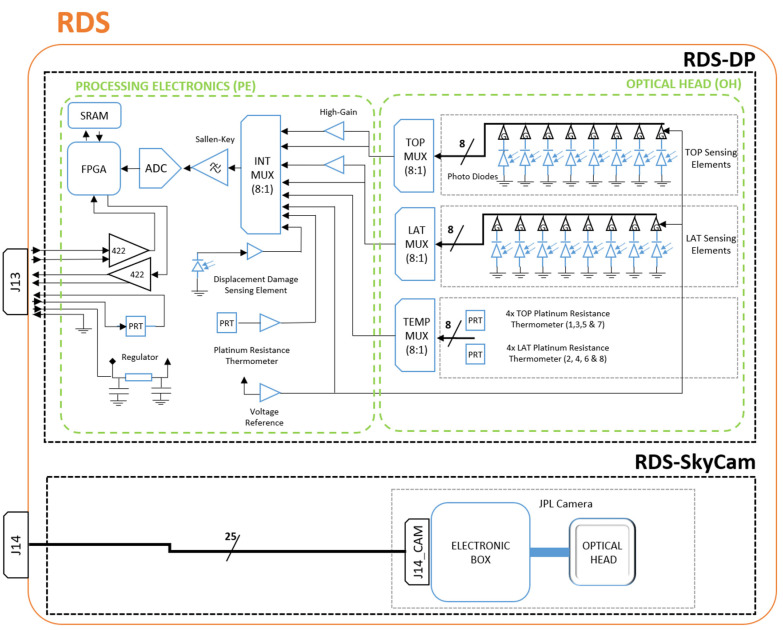
RDS, electrical, and electronics scheme.

PE board (Figure 6(2)) definitely has more space; it has a 77 × 95 mm^2^ area, enough to include all of the needed electronics to provide RDS-DP processing, storage, and communication capabilities. The analog signals from OH are distributed in a final multiplexor along with the rest of signals coming from PE: (i) internal voltage levels as housekeeping; (ii) post-amplified OH optical channels for cases with low-level signals (twilight); (iii) internal “displacement damage sensor” dark current and its temperature that offer an estimation of the radiation received by the internal electronics [25]. Finally, the analog chain passes through a low pass Sallen-Key second-order filter before arriving at the 16-bit ADC (Figure 7).

An anti-fuse FPGA implements the state machine that controls RDS-DP behavior. The RDS-DP contains 30 analog channels that are all digitalized for each sensor acquisition. An oversampling scheme is implemented for which the number of samples to be taken for each of the 30 channels is configurable. This number of measurements is accumulated and stored in the telemetry. The averaging is performed on Earth with double-floating point precision instead of performing it inside the FPGA with integers. At the same time, the FPGA calculates the median absolute deviation (MAD), which is a low-resource implementation within the component, to infer the noise level of each channel during the acquisition and accumulation. A 128k × 8 bits SRAM is used to store the intermediate calculations and construct the telemetry to be sent to the ICU (MEDA’s computer).

A 2 MHz oscillator provides the primary clock source for the FPGA. This low value balances the speed of the system versus the reduced power consumption required. However, it is not enough speed to maximize the ADC throughput of 200 ksps (2.5 MHz clock). For this reason, the ADC is configured as a master providing its internal oscillator as a second clock source to the FPGA. This configuration improves the system performance, achieving a 1 Hz sampling rate (all the channels’ acquisition) with 1024 accumulations per channel.

RDS-DP uses a dedicated RS-422 differential serial link to communicate with the ICU. The master-to-slave protocol implements two different measuring commands. The nominal acquisition command requests to RDS is a complete measurement of all its channels with the number of samples to do that as a parameter. The high-gain acquisition command obtains the same telemetry as the nominal, but with an extra 40 gain factor applied to the optical channels. Another set of commands has been included for diagnostic purposes if the regular operation fails. For example, they permit the measurement of one individual channel without using the memory. Also, they permit testing the memory in different ways, which will be helpful if problems appear during operation.

Only one kind of telemetry is generated when the channels’ acquisition process ends. It contains the package’s header, the 30 channels’ value (32 bit words per channel), its pseudo-standard deviation (8 bit for each one), and the accumulated number parameter that was configured. In total, an RDS-DP measurement generates a 163 bytes telemetry package.

Concerning EEE parts, RDS-DP has been designed, in general, with Hi-rel space-grade components compatible with the mission space radiation requirements. Only the photodetectors and the gain resistors greater than 100 M Ohm were commercial off-the-shelf components (COTS) due to the lack of space-grade equivalents. Screening and qualification campaigns were done for those parts. The results demonstrated the components’ compatibility with the mission reliability requirements (NASA EEE-INST-002 Level 2 components) defined for rover’s payloads and non-critical systems.

#### 2.3.3. Design for Extreme Temperatures

Besides the typical spacecraft design considerations—launch vibrations, pyro-shocks, vacuum, and radiation—Mars missions are very demanding in regard to materials, processes, and parts due to the extreme temperatures that the hardware will suffer on the planet’s surface. The significant temperature difference between night and day and the accumulated cycles implies a significant reliability challenge.

**Figure 8:**
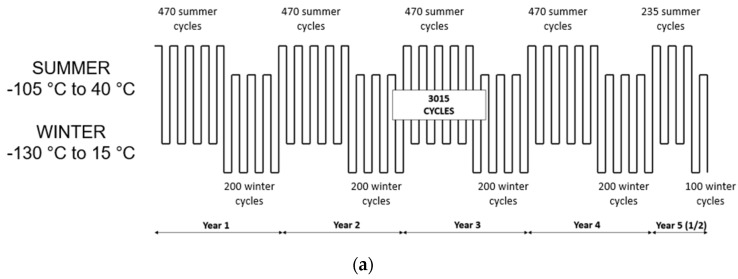

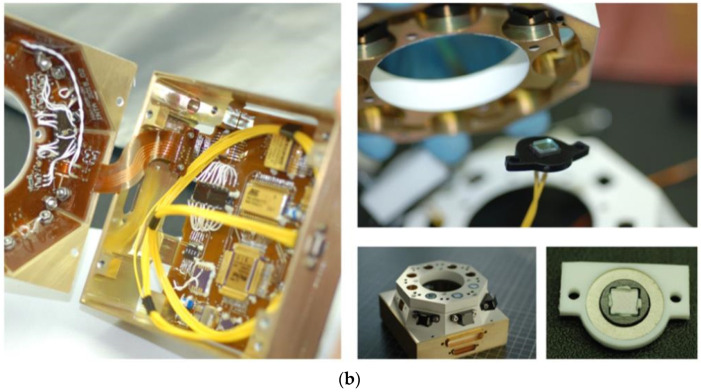
(**a**) PQV cycles description (3 × 1.5 MY); (**b**) RDS PQV assemblies under test.

Since the MER missions [26], NASA has applied a specific reliability test (Package Qualification and Verification—PQV), that qualifies the hardware for thermal cycling at large temperature ranges. On the MARS 2020 mission, subassemblies exposed to large temperature variations and assessed to be vulnerable required special PQV testing. For the RDS, the test consisted of subjecting the hardware to three times the mission’s duration cycles (1.5 MY) at qualification temperature ranges (for summer and winter sols definition), as shown in Figure 8.

Therefore, all RDS materials, processes, EEE components, and assemblies have passed a PQV test. A considerable qualification campaign entailed 15 active electronic parts, 5 unitary sensors (photodiode sets), 2 PCB materials, 1 thermal coating, 3 types of low outgassing glues, and 2 types of paint. The qualification depends not only on the materials or parts themselves. It also depends on how they are mounted or used. For that reason, INTA (Instituto Nacional de Técnica Aeroespacial) started the test by defining processes to apply to each board under test (BUT). They concerned the materials to use and how to use them (paints, glues, solders, and coatings.), the design of new footprints for components and how to solder them, and the definition of non-conventional component mounting styles, like flip-chip. More than 20 BUTs were manufactured to test all these technologies.

In addition, two different representative RDS-DP assemblies were manufactured be tested under PQV conditions. The first was a representative RDS model using a PCB almost equal to the final flight model (OH + PE). Meanwhile, the second assembly was based only on pigtailed photodetectors connected to the RDS interface connector without internal electronics. This was the plan-B solution if the test on the active electronics failed. The two options included three optomechanical sets each, to be validated as well.

The hardware under test was visually inspected at the beginning of the test. The major likelihood of failure due to thermal cycling should happen in the first hundreds of cycles. We performed 10 verification stops during the first simulated year on Mars and relaxed the next year’s frequency, lengthening to twice per year with one during winter cycles and another during summer cycles. During these verification stops, we performed the following steps: (i) a controlled cycle taking online measurements of all the BUT (the optical channels were checked using reference light sources inside the chamber); (ii) a visual inspection of all the systems under test; (iii) the optical systems were extracted from the chamber and transferred to an optoelectronics laboratory to test them for responsivity and angular response.

Most of the devices tested during this long campaign passed the test successfully. Significant concerns were related to the white paint used that broke and piled off after the first 50 cycles. After problem analysis, two different potential causes were identified. The first one was related to the drying time after painting, which was not appropriately followed. The second one was related to the temperature rate used during the test. To speed up the PQV, the test was performed at the maximum speed allowed (5 °C/min) and could be too stressful for the paint while being unrealistic when compared to the real situation on Mars. A second specific PQV was then performed on the white paint modifying these two parameters: the process and the test gradient offered optimal behavior.

### 2.4. RDS SkyCam

**Figure 9:**
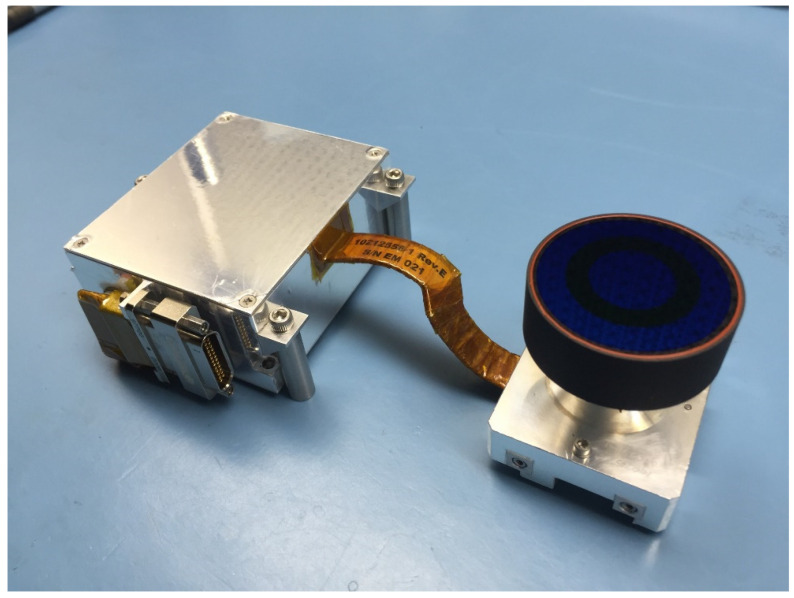
RDS-SkyCam flight model (FM) pre-integrated in the RDS structure.

The RDS-SkyCam (Figure 9) exists to make operationally simple measurements of dust size and shape in the Martian atmosphere several times per day by plotting the intensity of the sky as a function of angle from the Sun. The angular resolution requirement is 1 pixel per degree with a field of view requirement of at least 120 degrees. It is important that SkyCam correctly represents the sky intensity, which led to a new optical design. An additional requirement is the ability to simultaneously image the Sun and sky for at least a 1 h window, which requires a unique filter.

The SkyCam design heavily leverages the MER [27] and MSL HazCam [28], which have a 180-degree wide field of view across the diagonal, or a 124-degree center circular field. The flight electronics and detector were inherited spares from MSL, and the flight spare and engineering models electronics and detectors were inherited spares from MER. 

Furthermore, lessons learned from MSL showed significant debris and rocks on the top surface of the rover after landing, which led to the inclusion of a sapphire window on the RDS top plate to protect SkyCam.

#### 2.4.1. Optomechanical Design

The inherited HazCam optical prescription on the three powered elements was kept identical. However, the barrel, baffles, and other optical elements were redesigned to minimize stray light when the Sun is in the field of view or near the field of view. The RDS top cover window confined the field of view to a little more than a 124-degree full cone, which was good to eliminate direct sunlight from reaching the optic for morning and evening measurements of sky brightness. The sapphire window chosen for the RDS top cover provides mechanical protection without affecting the ability to make accurate measurements. Dust on the window should almost be resolved owing to the short focal distance of the fisheye lens.

**Figure 10:**
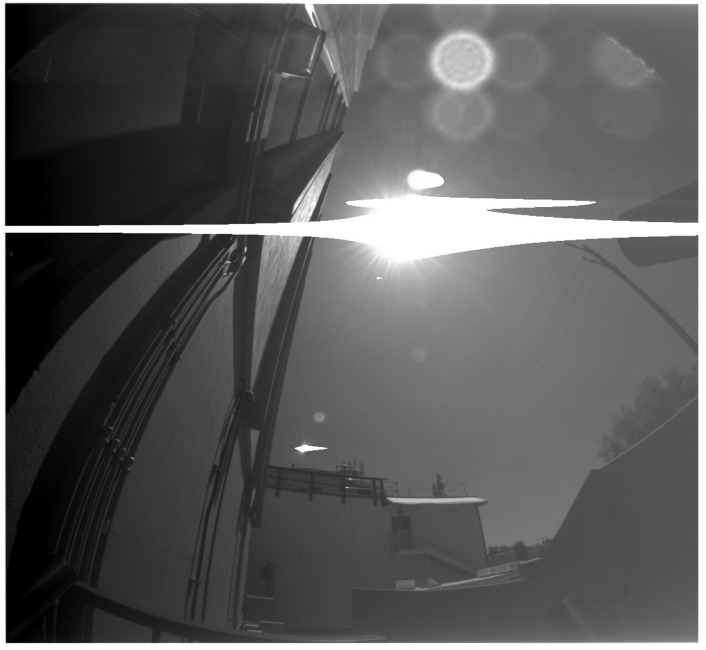
Outdoor testing of MER EM HazCam shows need for optical redesign for RDS-SkyCam.

The inherited HazCam optic was originally designed to mostly point at the ground without the Sun in the field of view, and it was not designed to make accurate radiance measurements. Upon inheriting the MER/MSL HazCam optical design, an outdoor test of an EM camera was performed as a quick validation of performance when imaging the Earth sky. As can be seen in Figure 10, there is significant stray light and ghosting that makes determination of sky brightness impossible. Further laboratory testing was performed during colorization of the InSight ICC camera (also a MER/MSL inherited HazCam), which proved that a bright illumination source outside the field of view led to significant stray light from internal reflections.

**Figure 11:**
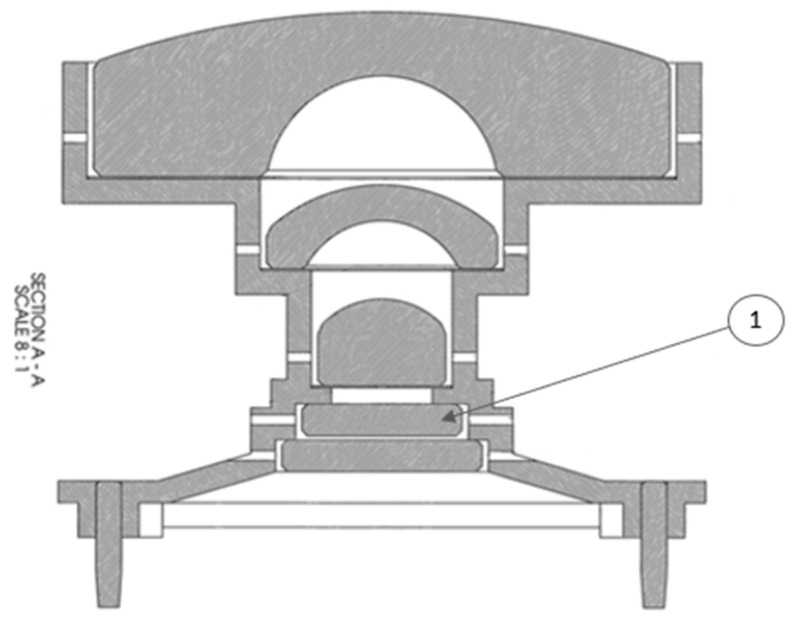
MER HazCam has a reflective ND1.1 filter on element 1, causing significant unwanted internal reflections.

**Figure 12:**
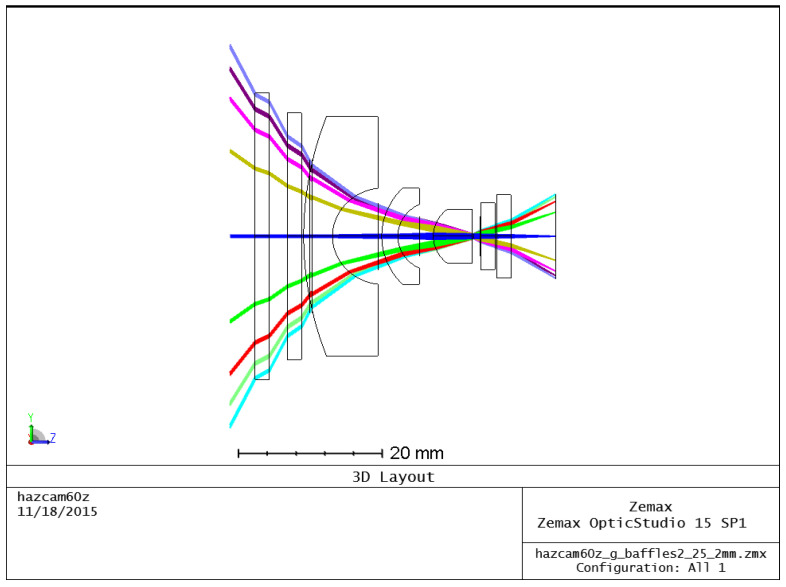
Raytrace of improved RDS-SkyCam optic through the RDS top cover sapphire window and new absorbing ND1.1 first element.

Analysis of the test results determined that an internal, reflective ND1.1 element caused significant internal ghosts (Figure 11(1)). The solution was to reconfigure the optic to have an absorbing ND1.1 element as the first element (Figure 12). This required modification of the barrel and an additional baffle. The requirement to add an absorbing ND5 filter to allow direct imaging of the Sun and Martian sky was realized as an absorbing ND5 annulus coating on top of the ND1.1 filter.

**Figure 13:**
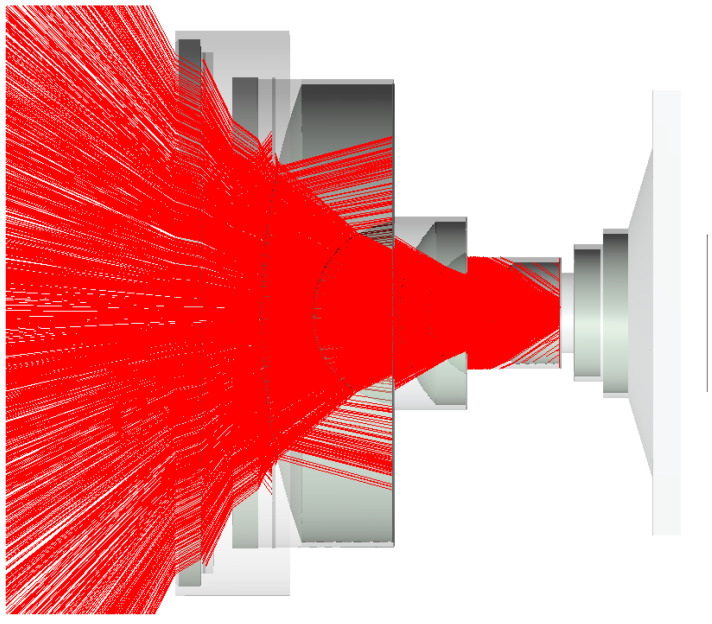
One million simulated rays using FRED aided in redesigning baffles and internal paint.

Further improvements were made to the optics by performing significant raytracing in FRED software (Photon Engineering), Figure 13, maximizing the size of the internal baffles, and ensuring all allowable internal surfaces were painted with Aeroglaze Z306.

**Figure 14:**
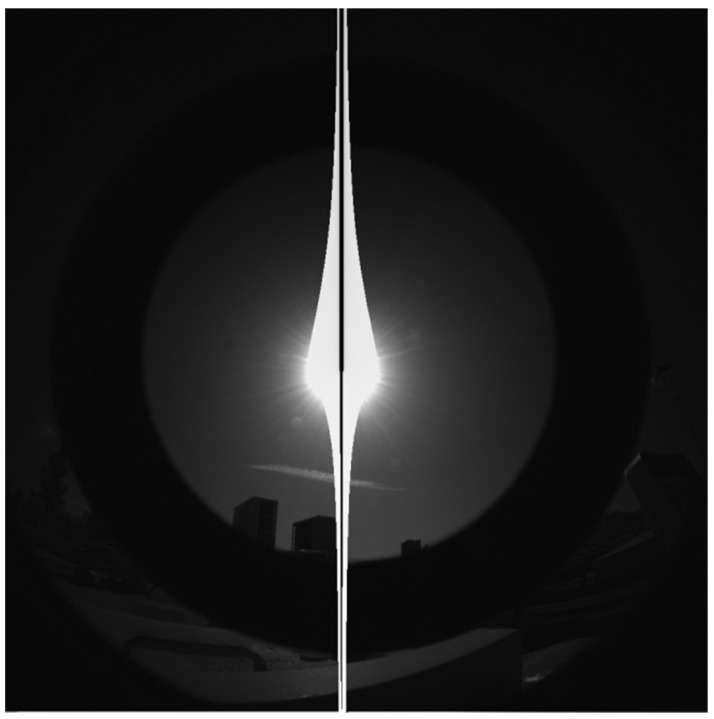
RDS-SkyCam image of Earth sky with new optic. Significant improvement over inherited design.

**Figure 15:**
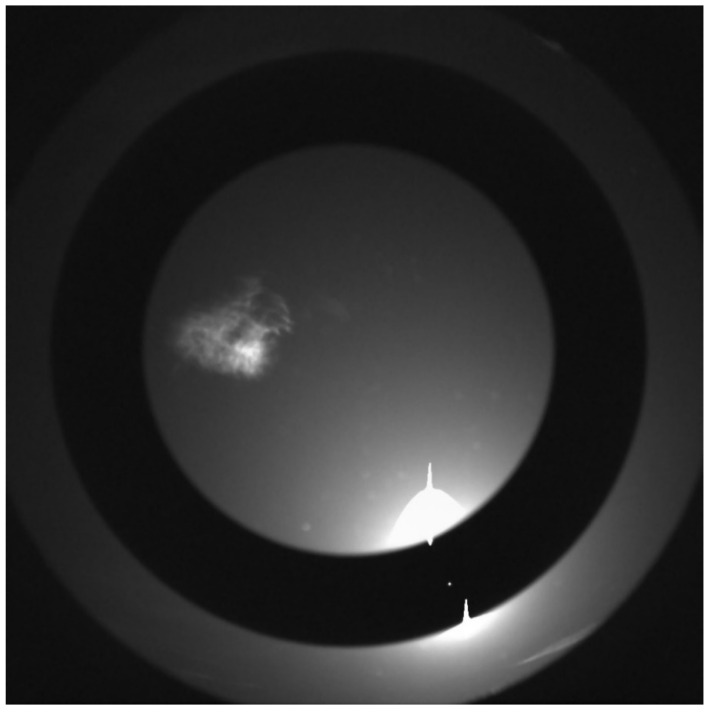
RDS-SkyCam image of Earth sky with new optic. Sun is visible through the ND5 filter while measurements of the sky brightness are simultaneously possible.

The optical design changes were qualitative verified on an engineering mode of the redesigned lens. No ghosts or other significant unwanted optical effects were observed with the sun is in the center of the field of view (Figure 14). The black line in the center is due to a zero-second exposure subtraction and there is a cirrus cloud below the sun. A few specs of dust on the lens show up as small, unfocused bright circles. On Mars, the RDS protective sapphire lens is a further distance from the detector, and any dust on the the sapphire surface will have a smaller radius. Figure 15 validates that the sun can be measured through the ND5 annular ring while the sky intensity is simultaneously within a measurable range, even though the scattered light from dust in the Los Angeles atmosphere causes the intensity of the sky to exceed measurable range within a few degrees of the sun.

#### 2.4.2. Electronics Design

The MEDA ICU was designed to support the RDS-SkyCam legacy interface as is [27]. The camera consumes 2.5 Watts from −10 and +5 V interfaces. The legacy data interface is simple LVDS with no high-level protocols or error correction. While the original MER/MSL cameras used a 10 MHz clock, MEDA-SkyCam uses 9.6 MHz for communication and operation.

#### 2.4.3. Thermal Design

The camera electronics have a wide allowable, non-operational flight temperature of −120 °C to +50 °C. The RDS mechanical structure was designed to avoid changes from the inherited MER/MSL design. The camera head has a PRT but no heaters since it can operate between −120 °C and +50 °C. Colder temperatures are preferred for the CCD, and the thermal noise makes imaging above 35 °C of questionable value.

However, the operational temperature of the electronic box of the camera was −55 °C to 50 °C, and therefore, the internal heater was increased to be compatible with the Jezero environment, from 2.5 W to 3.5 W.

### 2.5. RDS Final Figures

**Figure 16:**
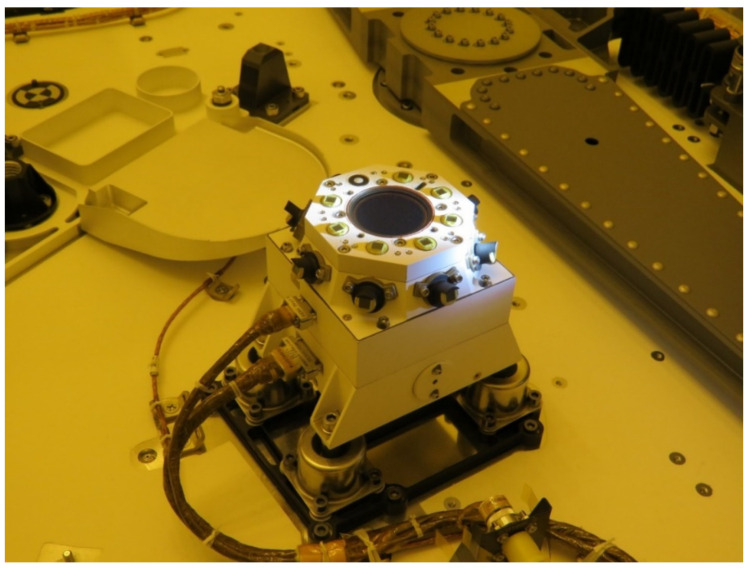
Final visual inspection of the RDS integrated on the Perseverance Rover.

RDS’s final design, summarized in Table 1, results in a compact instrument around 1 kg in a quasi-cubic volume of 100 mm of side. In Figure 16 the two different detecting technologies can be seen, each with their own electrical interface. 

## 3. Integration and Qualification

The internal camera did affect not only the sensor design but also the integration process. The RDS-SkyCam did not impose stricter requirements than RDS-DP (A-300) in terms of particulate and molecular contamination. However, its design was not compatible with the DHMR (dry heat microbial reduction) sterilization process. This complicated the integration as it was made necessary to take numerous biological assays between integration steps where last-access volumes were generated. In total, 50 swabs were done during the integration process divided into 3 steps; each step involved a three-day waiting time to know if the areas to be subsequently closed were clean or not. An HEPA (high-efficiency particulate air) filter was included into the RDS’ venting hole, increasing the internal bioburden density requirement (Figure 3(2)) from 300 to 1000 spores/m^2^, which was easier to achieve. Nevertheless, external surfaces shall have, in any case, less than 300 spores/m^2^ at the delivery.

The qualification and flight models of RDS passed the corresponding qualification and acceptance test levels according to the mission requirements. These tests included random vibration, quasi-static loads, thermal-vacuum, and pyro-shock SRS (shock response spectrum) tests. The inclusion of shock absorbers introduced particularities during this campaign because they could over- or under-test the RDS itself at random loads. On the one hand, the absorbers introduced a low-frequency mode around 160 Hz for the z-axis and 60 Hz for in-plane axes; on the other hand, high frequencies were dumped from those modes (isolation typically begins at frequencies beyond $\sqrt{2}*Natural\;frequency$) up to 2k Hz (top limit for random vibration test).

**Figure 17:**
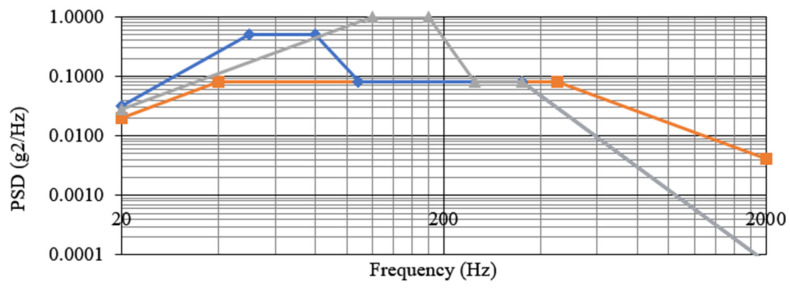
Random vibration profiles comparison. The orange line represents the initial requirement before isolation and the final profile performed over the shock absorbers with the dummy. The blue line represents the new profile for RDS-alone z-axis testing. Greyline represents the new profile for in-plane testing.

For that reason, JPL agreed to split the qualification campaign and test the RDS and the shock absorbers separately. The first step was a previous characterization of the absorbers with the STM (structural and thermal model) to infer the qualification’s correct levels. Then RDS was tested with its own load levels (Figure 17). The shock absorbers were tested alone, too, using an RDS dummy (mass and envelope representative).

INTA’s Dornier Table shock facility could not achieve 6000 g for the pyro SRS shock test; thus, a particular characterization was done for the absorbers. They were first studied individually with ¼ of the RDS mass. Later on, a modification of one free-fall INTA’s facility allowed testing the whole system at the needed 6000 g SRS. The modification consisted of removing the damping material at the end of the tower, permitting a metal-to-metal shock that met the pyro shock test’s time and slope requirements. The results with STM concluded that shock absorbers reduce the actual SRS profile reaching RDS from 6000 g to 400 g for frequencies beyond 400 Hz. Therefore, the qualification campaign was again split out into two different tests. The QM (qualified model) shock absorbers were qualified in the free-fall tower with an RDS dummy installed for the 6000 g SRS level test. The RDS QM was qualified with a 2000 g SRS test at the Dornier Table.

Verifying the RDS during the qualification and acceptance campaigns required a complete optical characterization before and after the whole test campaign. This included responsivity and angular response function verification (same test as the calibration). Additionally, functional tests and reduced optical characterizations were carried out between tests or even within tests (e.g., different axes of a vibration test) with the same goal.

## 4. Sensor Calibration and Performance

RDS has taken the heritage from previous developments not only for its design but also for its calibration process. The method [29] developed for the calibration of the DREAMS-SIS sensor was followed and improved for the RDS discrete photodetectors. In the camera case, the process was divided into two steps. The first was to calibrate the camera alone, with the new optics designed for the RDS, following the same method used for MERS and MSL engineering cameras [30,31,32]. The second consisted of a set of tests performed in the final RDS configuration. This system-level test allowed for evaluating the impact of the optical path elements, top housing baffle, and protection sapphire by means of the camera’s final behavior.

### 4.1. RDS-DP Calibration

Calibration activities for RDS-DP were carried out at the Space Solar Cell Testing Laboratory—SPASOLAB—situated at INTA’s campus. This is an official laboratory for testing solar cells according to ESA standards, with several sun simulators and Xenon lamps. 

The RDS-DP model defines the current *I*(A) generated in a photodetector when it is exposed to a light source as:

(1) $$I\;\left(T,\alpha ,\lambda \right)=\int_{0}^{\infty }R\left(T,\;\alpha ,\;\lambda \right)\;E\left(\lambda \right)d\lambda$$

where *E* is the spectral irradiance (W m^−2^ nm^−1^) received by the photodetector and *R* is the throughput (A m^2^ W^−1^) which depends on the temperature (*T*), the incidence angle (*α*), and the wavelength (*λ*). The throughput can be expressed as $R\left(T,\;\alpha ,\;\lambda \right)=ARF\left(\alpha \right)\cdot TRF\left(T\right)\cdot r\left(\lambda \right)$, where *ARF* (angular response function) is the sensor angular response, taking values between 0 and 1, *TRF* (temperature response function) represents thermal dependence, and *r*(*λ*) is the spectral response of the detector (filters + photodiode). From these functions, we have:

(2) $$I\;\left(T,\alpha ,\lambda \;\right)=ARF\left(\alpha \right)\cdot TRF\left(T\right)\;\int_{0}^{\infty }r\left(\lambda \right)\;E\left(\lambda \right)d\lambda$$

The last term of the right side of Equation (2) can be simplified if the calibration is performed with a solar simulator and defines a mean resposivity $R_{\lambda_{1}}^{\lambda_{2}}$ for each corresponding channel as:

(3) $$R_{\lambda_{1}}^{\lambda_{2}}=\frac{\int_{0}^{\infty }r\left(\lambda \right)\;E\left(\lambda \right)d\lambda }{\int_{\lambda_{1}}^{\lambda_{2}}E\left(\lambda \right)d\lambda }=\frac{\int_{0}^{\infty }r\left(\lambda \right)\;E\left(\lambda \right)d\lambda }{E_{\lambda_{1}}^{\lambda_{2}}}$$

where *λ*_1_ to *λ*_2_ are the wavelengths defining the passband of each channel, and $E_{\lambda_{1}}^{\lambda_{2}}$ is the irradiance (W m^−2^) in the range [*λ*_1_, *λ*_2_]. Therefore, with a Sun spectrum simulator, we can express the current at each channel as:

(4) $$I\;\left(T,\alpha ,\lambda \;\right)=ARF\left(\alpha \right)\cdot TRF\left(T\right)\cdot R_{\lambda_{1}\mathrm{sun}}^{\lambda_{2}}\cdot E_{\lambda_{1}\mathrm{sun}}^{\lambda_{2}}$$

Finally, it is necessary to introduce an *offset* that results from the bias generated by the electronics and the dark current from the photodetectors. With this *offset*, which usually depends on the temperature, Equation (4) becomes:

(5) $$I\;\left(T,\alpha ,E_{\mathrm{sun}}\;\right)=ARF\left(\alpha \right)\cdot TRF\left(T\right)\cdot R_{\lambda_{1}\mathrm{sun}}^{\lambda_{2}}\cdot E_{\lambda_{1}\mathrm{sun}}^{\lambda_{2}}+Offset\left(T\right)$$

**Figure 18:**
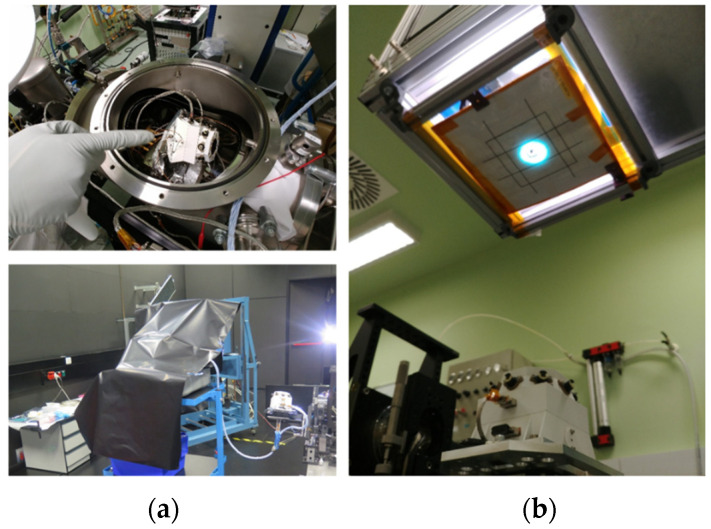
(**a**) On top, the RDS offset and *TRF* calibration set-up. On the bottom, the RDS angular calibration. (**b**) RDS responsivity calibration.

With this scheme, the calibration procedure of RDS-DP was done in the following steps (Figure 18):

1. Offset calibration.
2. Temperature response function calibration.
3. Responsivity calibration.
4. Angular response function calibration.

The final RDS-DPperformance is summarized in Table 2. We can observe that only one parameter is out of the specification. The TOP7 dynamic range was underestimated due to an incorrect characterization of the diffuser that was used on the upper part of this optomechanical assembly. Considering that (i) the substitution of the corresponding resistors in the amplification gain was a hazardous operation, (ii) the high impact of this operation on the project schedule, and (iii) the rationale that for only a short period of the year the channel would be saturated (around 60 days per martian year, 2 h saturated in the worst day case) and its information would be derived by the rest of the TOP channels, it was decided to use the RDS flight model “as is”.

### 4.2. RDS-SkyCam Calibration

Calibration activities for RDS-Skycam were carried out in two major phases, one at JPL and one at INTA in SPASOLAB. Initial tests at JPL confirmed that CCD performance was in keeping with other MER and MSL CCDs. The camera response is linear to within 2 data numbers (DN) for signals below 300 DN and to within 1% over the 300–3000 DN range. The gain is 55 electrons/DN, and the read noise is 30 electrons, while full well is 160,000 electrons. As with similar CCDs, blooming occurs for large signals; this produces primarily column-wise spreading of saturated pixels but can spread row-wise for the largest signals, such as solar imaging without the neutral density filter.

Focal plane assembly bias and CCD dark current calibration was split across both sites, with thermal calibration at INTA. For typical operational settings, the electronics bias was determined to be <10 DN at temperatures <−40 °C, but rise above 60 DN at 40 °C. The frame transfer dark current, which occurs after the image has been transferred to an optically non-active part of the array and as it is being read out, is <1 DN at −20 °C and rises to 200 DN at 40 °C (for the last two rows read out). The active dark current, which is accumulated during the camera exposure, ranges from 0.1 DN/s at −40 °C to 300 DN/s at 40 °C. For operational temperatures and typical exposures of 0.8 s (mid-sol Sun images), 3 s (morning and afternoon sky images, and 10 s (near sunrise or sunset images), mean dark current is expected to vary from 1–60 DN. Dark current is expected to be correctable to within a few DN for pixels that have not changed their behavior due to (for instance) cosmic ray strikes or RT radiation; such ‘hot’ pixels will be filtered out in data analysis.

Room temperature tests at JPL also measured the camera’s geometric performance, flat field, and absolute responsivity at room temperature. Geometric performance was in keeping with the MER and MSL equivalents with an optical resolution of 0.3–0.4 degrees (about 3 pixels, with 8.3 pixels/degree). The flat-field response was measured with a wide-angle integrating sphere and then again with overlapping images of an integrating sphere that did not fill the FoV in order to remove integrating sphere artifacts that appeared in the images due to the very close hyperfocal distance. The absolute responsivity of the central 100 × 100 pixels was determined to be 3.79 × 10^−5^ W m^−2^ sr^−1^ nm^−1^ (DN/s) at 23 °C. INTA tests showed that there was no measureable variation with temperature. The response falls to about 25% at the edges of the FoV.

At INTA, a solar source was imaged at varying angles to test the ND coating. By comparing frame-transfer images (0 s images that collect signal only as the CCD is shifted 1024 pixels in 5.2 ms) of the solar source in areas with a low-response outside the ND coating with longer exposure images of the solar source within the ND coating, it was determined that the ND coating caused 10^−5^ extinction, as planned. Such images were also used for stray light testing, verifying that the camera had met its stray light performance goals.

The final RDS-SkyCam performance is summarized in Table 3.

## 5. RDS Retrieval Procedures for Mars

### 5.1. Dust Characterization: Optical Depth, Phase Function, and Single Scattering Albedo

RDS-Skycam will be used to monitor optical depth due to dust and ice aerosols (gas scattering opacity is about 0.002 in the visible, >2 orders of magnitude less than expected values for aerosols). Images will be calibrated to radiance, then the Sun will be identified if it is within the ND area (with margin for vignetting) and its flux will be measured via synthetic aperture photometry with a local background rejection to combat residual stray light. Initial measurements may rely on the absolute response calibration. More typically, optical depth will be determined by solar extinction as obtained by a relative calibration on Mars (thus compensating for time-varying effects like dust on the optics). First, the Mars 2020 Mastcam-Z instrument opacities [33] will be determined from a relative calibration (e.g., [12]) using high-Sun and low-Sun imaging. Next, Skycam opacity will be normalized to match Matscam-Z opacity over nearby sols, as Skycam can only image the Sun at fixed elvation angles. Finally, that calibration will be applied to the full set of skycam solar images (expected to be a few times larger than the set of Mastcam-Z solar images).

Airborne dust properties can also be studied by comparing the time variation of the sky intensity measured by RDS-DP with radiative transfer simulations. The sky brightness on Mars mainly depends on the dust scattering properties and the vertical distribution of dust particles: the single-scattering albedo (*ω*), the phase function (*P*(*Θ*)), and the extension cross-section (C_ext_). These scattering parameters, required for the radiative transfer simulations, can be computed with a T-Matrix approach [34,35] (to account for the non-sphericity of dust particles) for known values of the dust size distribution (effective radius r_eff_ and effective variance v_eff_) and refractive index *n* (*n* = *m* + *ki*). 

**Figure 19:**
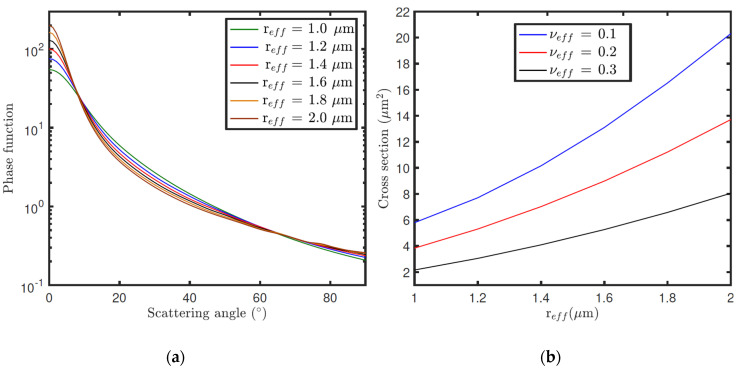
(**a**) Phase functions of equal cylindrical particles computed at 0.75 μm for a set of r_eff_ values and a constant v_eff_ = 0.3. (**b**) Variation of the equal-cylindrical-particle extension cross-section with r_eff_ at 0.75 μm for a set of v_eff_ values.

**Figure 20:**
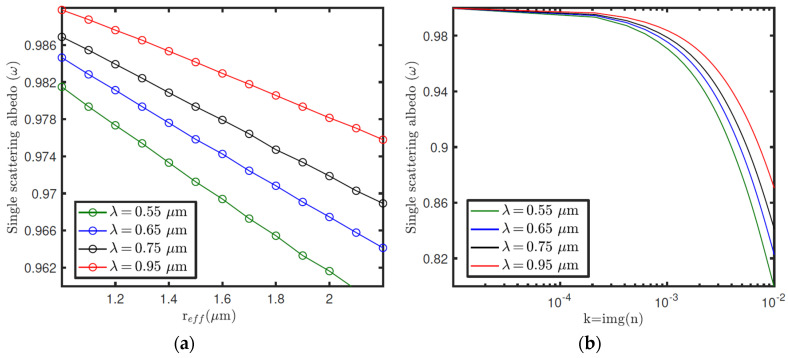
(**a**) Variation of the single scattering albedo with r_eff_ at different wavelengths and for constant values of veff and n. (**b**) Variation of the single scattering albedo with *k* = img(*n*) at different wavelengths, and for fixed values of r_eff_, v_eff_ and *m* = real(*n*).

Figure 19 shows, as an example, phase functions (Figure 19a) computed with a T-Matrix for equal cylindrical particles, for a set of r_eff_ values, and the variation of the extinction cross-section (Figure 19b) with r_eff_, for a set of v_eff_ values. Both parameters were computed for *λ* = 750 nm and *n* = 1.4 + 0.0001*i*. For a given v_eff_, larger particles result in greater cross-sections, and thus higher opacities (for a constant dust number density). We also observe that the greatest variations of the phase function with r_eff_ are given at scattering angles between 0° and 10°. Therefore, this indicates that the periods of the day for which the angles between the sun direction and the viewing direction of each sensor cover this range are suitable for estimating the dust size distribution. Although the phase function also depends on the refractive index, the variations in the sky brightness with n are mainly due to the impact of n on the single scattering albedo. Figure 20 illustrates the variation of the single scattering albedo with r_eff_ (Figure 20a) and *k* = imaginary(*n*) (Figure 20b) at different wavelengths. In this case, the larger are r_eff_ and *k*, and the smaller *ω* is for given values of v_eff_ and the real part of the refractive index (m). Similar computations were performed for the real part of the refractive index, but we did not find significant variations in the scattering parameters at RDS wavelengths for the range of m values expected on Mars.

**Figure 21:**
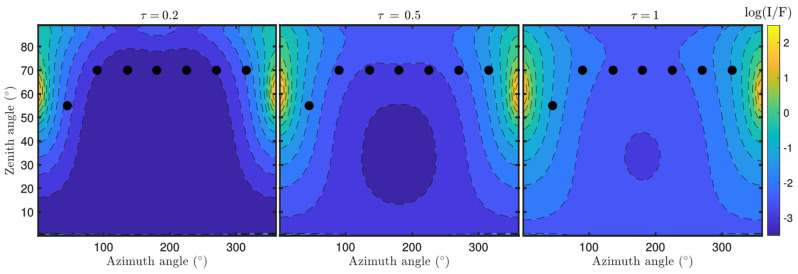
Variation of sky brightness with zenith and azimuth angles simulated for different values of dust opacity. Black dots indicate the angular positions of lateral RDS sensors. In addition to these observations, top channels will provide similar measurements but for a zenith angle ~0°.

**Figure 22:**
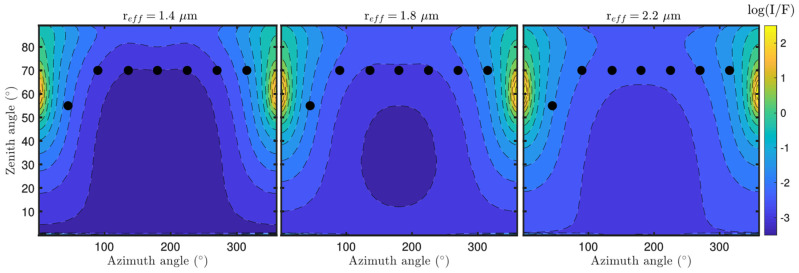
Same as Figure 21 but for different values of the effective radius r_eff_.

From the scattering properties and vertical distribution of dust number density (n_dust_), the sky brightness on Mars can be simulated with a radiative transfer code. Figure 21 shows the radiance factor (I/F) as a function of zenith and azimuth angles, simulated for three different dust opacities (τ). The black dots indicate the angular positions of RDS-LAT sensors for a given orientation of the instrument. These simulations were performed for a solar zenith angle (*SZA*) of 60°, and r_eff_, v_eff_ and n values of 1.4 μm, 0.3, and 1.4 + 0.001*i* (assumed to be the same at all vertical levels), respectively. For these simulations it was also assumed that the dust number density decreases exponentially with altitude with a scale height of 10 km, and where the dust opacity is computed from C_ext_ and n_dust_. Note that the changes of τ in Figure 21 are produced by variations in n_dust_ as r_eff_, v_eff_ and n, and so C_ext_, are fixed to a constant value. From these simulations, we observe that the magnitude and angular distribution of the sky intensity largely depend on τ. Similar simulations but for r_eff_ are illustrated in Figure 22. In this case, an increase in r_eff_ results in variations in *P*(*Θ*) (Figure 19a) as well as in a greater value of C_ext_, and so of τ (Figure 19b). Therefore, the size distribution and refractive index of dust particles, needed for computing the dust scattering properties, can be inferred by searching the values of these parameters that provide the best fit between the simulations and the observations made by the sensors [36,37].

**Figure 23:**
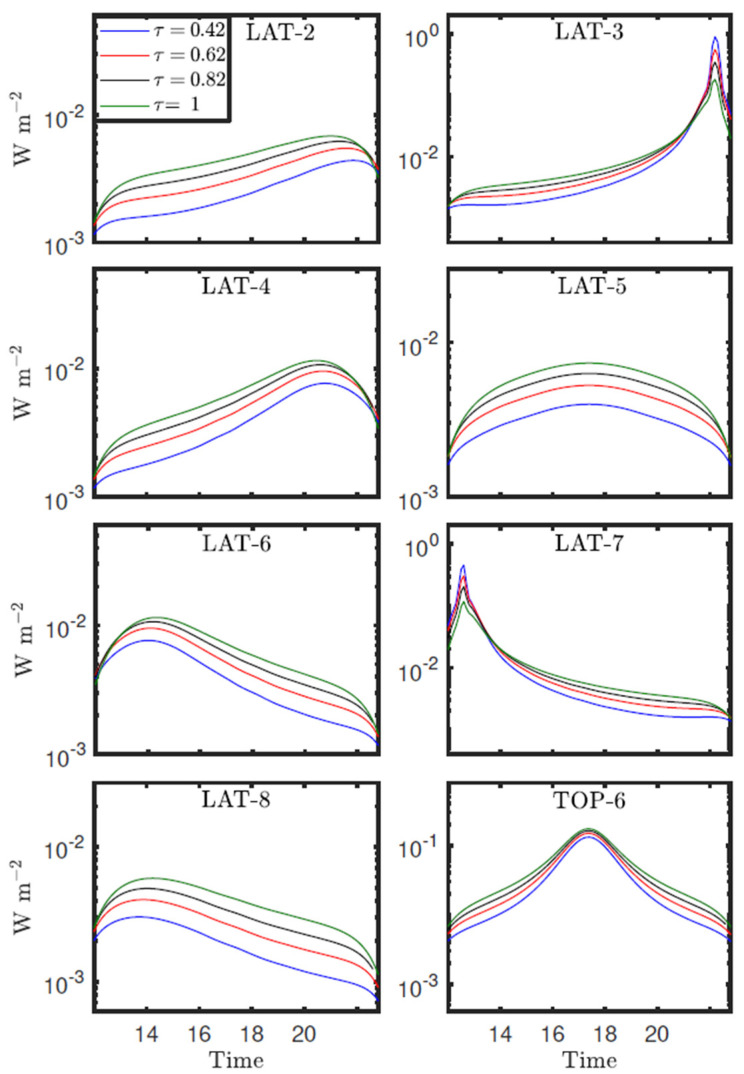
Simulated daytime RDS lateral and TOP-6 sensors output intensities for different dust opacities (or number densities). The rest of the dust parameters were set to r_eff_ = 1.25 μm, v_eff_ = 0.22, and *n* = 1.5 + 0.0004*i*.

For searching the optimal values of the dust parameters, first we must simulate the RDS signals. For given values of the dust parameters, we can simulate RDS signals during the day using Equation (5) (Section 4), where the solar irradiance $E_{\lambda_{1}}^{\lambda_{2}}$(W m^−2^) is computed integrating the intensity field (computed with the radiative transfer code) over the field of view (FoV) of each sensor. Figure 23 illustrates $E_{\lambda_{1}}^{\lambda_{2}}$ signals for LATs and TOP-6 sensors simulated during the day for different dust opacities. These simulations indicate that the magnitude and shape of the different signals depend on the dust load in the atmosphere, and thus information on this parameter can be inferred from the observations and the simulations. 

Similar simulations demonstrate that RDS observations are sensitive to the rest of the dust parameters discussed above. However, the precision on which these parameters can be constrained depends largely on some other factors such as the sun elevation or the possibility to combine RDS-DP observations with Skycam images. For instance, the best characterization of the dust size distribution is done when observations at scattering angles ranging from 0° to 180° are available. These scattering angles can be achieved when the Sun is within or close to the FoV of one of the sensors, or by combining Skycam images with RDS-DP observations at different wavelengths. As RDS-DP and Skycam are not expected to operate continuously along the day, we must select the times (according to the day of the year and rover position) to better constrain the size distribution or refractive index of dust particles. 

### 5.2. Dust Lifting Events

Dust lifting events such as dust devils play a key role on the Martian dust cycle. For instance, dust devils are thought to account for the ~30–50% of the total dust budget [38], and they represent a continuous source of lifted dust, active even outside the dust storms season. In this regard, dust devils have been identified as one of the main mechanisms able to sustain the ever-observed dust haze of the Martian atmosphere. Information on the dust devils’ occurrence mainly comes from the observations made by cameras onboard orbiters or the NASA Mars rovers. Although these observations provide key information about these events, they do not cover the entire diurnal cycle for a significant number of days along the year (as imaging cannot be performed at high temporal resolution during the entire day). Furthermore, the cameras onboard orbiters are unable to detect small-scale dust lifting events. In contrast to imaging from cameras, photodiodes observations require relatively low power and data volume, and can be performed at high temporal resolution for long periods of time. In this regard, previous analyses have demonstrated the possibility for dust devil detection employing the REMS sensors [39]. However, as REMS sensors are pointed at the zenith, this detection is possible only for very close dust lifting events. Our radiative transfer simulations indicate that variations in the dust loading near the surface can be detected by RDS lateral sensors. Due to the 20° of elevation above the horizon, RDS-LAT detectors can be employed to detect dust devils at large distances (about three times the altitude of the event). Therefore, the future observations from RDS-LAT sensors on Mars offer a unique opportunity to monitor dust lifting events at high temporal resolution from sunrise to sunset, and with an excellent spatial coverage.

### 5.3. Cloud Detection, Cloud Optical Depth, and Height Determination

Cloud detection with RDS is carried out using Skycam images or RDS-TOP sensor observations at twilight. In this work ‘twilight’ is defined as the period for which *SZA* takes values between 86° and 98°. Cloud detection at twilight is carried out by looking at the time evolution of the color index (*CI*), defined as:

(6) $$CI\left(SZA\right)=\frac{I\left(\lambda_{1},SZA\right)}{I\left(\lambda_{2},SZA\right)}$$

where *I* is the measured intensity at zenith at wavelengths *λ*_1_ and *λ*_2_. When high clouds are present during twilight, they produce a maximum or a minimum (depending on the choice of *λ*_1_ and *λ*_2_ and the atmospheric properties) in the *CI* signal, whose *SZA* of maximum or minimum *CI* (*SZA_max_*) depends on the cloud altitude. This approach, that is based on the variation of the atmospheric transmission with *λ*, has been previously used and tested for the characterization of cirrus [40] and polar stratospheric clouds on Earth [41,42]. As for the dust properties’ estimation, the altitude of the clouds is derived using radiative-transfer simulations. The simulation of *CI* at twilight requires a radiative-transfer model in spherical geometry as the plane-parallel approximation is not valid for *SZA* > 80°. A representative model for a Martian-type cloud is a thin layer at a given altitude with a spatial distribution of cloud particle density characterized by a Gaussian height profile, which is scaled to produce the required cloud opacity. Therefore, once a cloud is detected by RDS-TOP channels, the altitude of the cloud can be inferred by comparing the *SZA_max_* with the values obtained by the simulations. 

### 5.4. Ozone Column Abundance

The characterization of ozone is important in Martian atmospheric photochemistry since odd-hydrogen species, produced from water vapor photolysis, compete for atomic oxygen involved in ozone production, and are also involved in the catalytic cycles governing ozone loss [43,44,45,46,47,48]. As a result, an anticorrelation between ozone and water vapor profiles appears in Mars’s atmosphere (e.g., [49,50]). These species, with abundances less than 1 part per billion in volume (ppbv), play fundamental roles in the long-term stability of the CO_2_-dominated atmosphere of Mars and, in general, have not been directly measured. Therefore, ozone becomes an important tracer for Martian photochemistry and is used for the validation of atmospheric models [45,46,48,51]. 

Although our knowledge of Mars’s ozone distribution and variability has been significantly improved with the arrival of several orbiters, it has never been measured from the surface, which could complement the data acquired from orbiters to characterize this specie.

MEDA RDS will attempt to measure the ozone column abundance from the Martian surface by means of differential absorption measurements in the Hartley band, which is challenging given the low abundance of ozone in the Martian atmosphere (less than 3 µm-atm in the latitudes enveloping the Mars 2020’s landing site region). In particular, the RDS includes two photodiodes, the first one in 255 ± 5 nm (where ozone presents strong absorption) and the other one in 295 ± 5 nm (where the ozone absorption is strongly reduced) (see Table 2). Radiative transfer modeling will assist in deriving ozone abundances, considering other species and aerosols present in the atmosphere. 

## 6. Terrestrial Field Campaigns

The performance of the terrestrial prototype of RDS was evaluated during two test campaigns conducted at the INTA/Atmospheric Observatory “el Arenosillo” (INTA/ARN) in southwestern Spain. This region is characterized by its high-aerosol-loading conditions during episodes of Saharan dust intrusions. A significant advantage of this location is the presence of a CIMEL sun photometer of the AERONET network to test RDS aerosol retrievals. In these campaings, however, the retrieval procedures for ozone (employing the UV channels) couldn’tbe tested as a result of the strong Hartley Band absorption.

### 6.1. RDS Measurements

**Figure 24:**
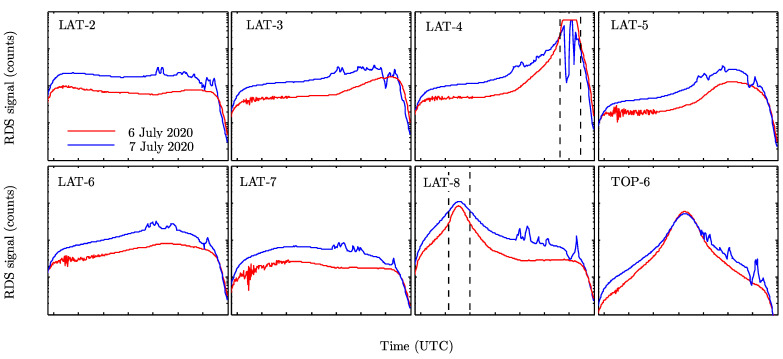
RDS LAT and TOP-6 sensors’ measurements in INTA/ARN on 6 (red solid line) and 7 (blue solid line) July 2020. The bands delimited by black dashed lines indicate the time intervals for which the Sun is near the sensors FoV.

The campaigns were carried out from 26 July to 2 August 2019, and from 6 to 8 July 2020. Only for the campaign from 6 to 8 July 2020 was Saharan dust intrusion recorded. Figure 24 shows the evolution of RDS LAT and TOP-6 sensors signals measured for two days of the campaign, 6–7 July 2020, in INTA/ARN. Due to a lack of the SkyCam EGSE (electronic ground support equipment) sky images were not available for these days. The bands delimited by vertical dashed lines represent the time intervals during which the Sun is near the FoV. The RDS sensors throughout the day can measure either only scattered sunlight (e.g., LAT-2 and LAT-3 in Figure 24) or scattered sunlight and direct light for some time intervals (e.g., LAT-4 and LAT-8 in Figure 24). By comparing the signals of both days, we observe sharp variations in the signals measured on 7 July after 12 UTC. These variations are produced by the presence of thick clouds within the FoV of the sensors, leading to fast variations in the signals. Therefore, only the cloud-free time intervals are selected for the dust properties analysis. We also observe that signals measured on 7 July are greater than those on 6 July, indicating a change in the sky brightness during these days. As will be discussed in the next section, these results are due to variations in the aerosols load during the campaign period.

**Figure 25:**
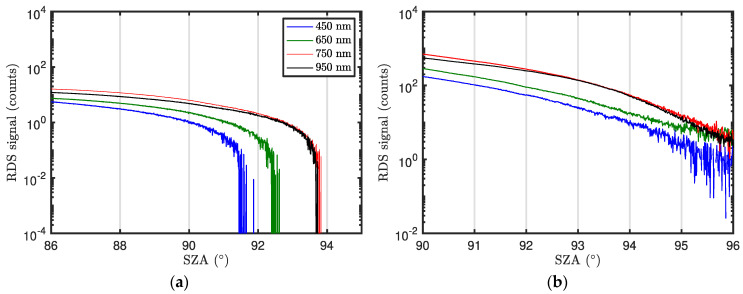
(**a**) RDS TOP-4 (450 nm), TOP-5 (650 nm), TOP-6 (750 nm), and TOP-8 (950 nm) measurements at twilight. (**b**) Same as left panel but using the second gain factor.

In addition to the daytime measurements, for each day of the campaign, RDS was set during the twilights for detecting high clouds using the CI. Three different CI signals were computed combining TOP-4 (*λ*_1_ = 450 nm) signal with TOP-5 (*λ*_2_ = 650 nm), TOP-6 (*λ*_2_ = 750 nm), and TOP-8 (*λ*_2_ = 950 nm) signals. However, CI signals did not show any maximum that may indicate the presence of high clouds during these time periods (this was also confirmed by cameras operating at INTA/ARN during the campaign). Figure 25a shows, as example, the signals measured by the TOP sensors at 450, 650, 750, and 950 nm. We observe that for *SZA* greater than ~91°, the second gain factor is required as the sky intensity is very low. Although the CI approach for detecting high clouds could not be tested during this campaign, the signals illustrated in Figure 25b indicate that RDS signal-to-noise at high *SZA*s remains high enough to perform these kinds of analyses. 

### 6.2. RDS-DP Dust Retrievals

**Figure 26:**
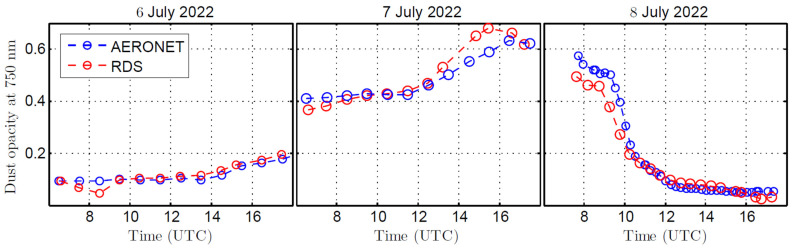
Aerosol optical depth derived from RDS observations and AERONET at 750 nm for the 3-day campaign period.

For the analysis of the dust properties discussed in Section 5.1, RDS signals measured during the 2020 campaign at INTA/ARN were fitted with our radiative transfer and retrieval code adapted to Earth atmosphere instead of Mars. The dust opacity at 750 nm was estimated at 1 h time intervals in order to record short-time changes in the dust concentration. Figure 26 shows the time series of dust opacity estimated from RDS observations, along with the values given by the AERONET photometer. Some data are missing because of the presence of thick clouds for some time periods over the course of the day. These results indicate remarkable increases in the dust opacity during the night for 6 and afternoon for 7, recording the highest values at around 16 UTC. For the last day of the campaign, we observed a sharp decrease in the dust opacity at around 9 UTC, indicating the dust intrusion at INTA/ARN lasted ~24 h. Note that for the last day, the dust opacity was derived at 30 min time intervals as a result of the fast variations in the atmospheric dust load. The comparison between RDS and AERONET dust opacity retrievals indicate that both instruments are in agreement with a correlation coefficient > 0.90. Some discrepancies are found at around 15 UTC on 7 July, produced by the presence of thick clouds during this time. In addition to the results shown on Figure 26, the dust phase function was retrieved from the observation made by the lateral sensors. As discussed in Section 5.1, the characterization of dust particle radius (or the phase function) is done at the times when the Sun is near to one of the lateral sensors’ FoV and the sky is free of clouds. These conditions were only met during the morning of 7 July (at around 9 UTC). Our analysis provided an effective radius r_eff_ = 1.96 μm, a value very close to the 1.99 μm derived by AERONET. The lack of Skycam images during this campaign limited the full characterization of the dust optical properties (e.g., estimate the refractive index). However, this preliminary campaign allowed us to evaluate the performance of RDS and compare its retrieval products against an AERONET photometer. 

## 7. Conclusions

During the last decade, INTA has worked on the design and development of miniaturized radiometers for Mars exploration. The radiation and dust sensor and its sibling, the solar irradiance sensor for ExoMars 2022, represent the last step of a long roadmap established by the institution. In this work we describe the different procedures adopted to develop an instrument which is capable of addressing a number of atmospheric scientific objectives while meeting the mission requirements, such as weight, power consumption, and size. To achieve all the scientific goals, RDS combines two different measurement technologies: multi-wavelength and different-pointing-orientation photodetectors, and one zenith camera looking directly to the sky. This architecture allows us (i) to better characterize the optical and scattering properties of suspended dust; (ii) to detect dust lifting events such as dust devils with a high sampling frequency and for long periods of time; (iii) to detect and characterize clouds (e.g., cloud altitude) during twilight; and (iv) to estimate, for the first time, the ozone column abundance from the Martian surface. All these scientific products will be generated for long periods of time, and thus as a function of season and local time. 

The complex design process of the RDS has been described in detail in this work with emphasis on the miniaturization, performance and power consumption requirements which were essential during this process. In this respect, JPL and INTA heritage from previous missions was crucial. On top of that, the reliability of the final instrument is a big challenge due to the extreme low temperature conditions and the wide thermal cycling in the mission environment. This was addressed by a long and detailed test campaign to qualify all the hardware involved in the RDS manufacturing (except for the camera, which was already qualified in the MER and MLS missions), and whose results are key for INTA’s future instrumentation developments. The flight unit integration, qualification, and calibration were performed while meeting strict planetary protection requirements, which made all the different processes more complex but ensured that the hardware would not contaminate the future samples acquired by Perseverance.

The instrument calibration process was carried out continuously verifying the scientific requirements of the uncertainty in the measurements. This was checked against the different retrieval models of each scientific investigation, as well as by the different terrestrial campaigns conducted with the RDS. For the terrestrial campaigns, the retrieval procedures were adapted to Earth conditions, and the results were tested during two field campaigns in “el Arenosillo” station (Spain), a location characterized by frequent dust events. During a Saharan dust intrusion, RDS retrievals were compared with an AERONET photometer operating at the same station. This comparison allowed us to verify the expected performance of RDS, as well as to evaluate the capabilities of the different retrieval models.

## Figures and Tables

**Table 1:** RDS design final figures.

Parameter	Value	Observations
Dimensions	RDS: 132 × 102.25 × 102.40 mm^3^ RDS + SA ^1^:	Maximum Envelope including fixation points volume.
Mass	RDS: 1042.60 g RDS + SA ^1^: 1320 g	
Power Consumption	RDS-DP: 75–59 mA @ 5.2 V RDS-Skycam: <400 mA @ 7 V <200 mA @ −10 V	For qualification temperature range and different SkyCam operational modes (without heating).
Temperature	RDS-DP: −128 °C–50 °C ^2^ RDS-Skycam: −128 °C–50 °C ^2^ −50 °C–50 °C ^3^	
Heater	3.5 W	Only for Skycam E-box subassembly
Interface	RDS-DP: RS-422 @ 57.6 kbps RDS-SkyCam: dedicated SPI 9.6 MHz (CLK).	Proprietary, character-oriented protocol
Data volume	RDS-DP: 163 bytes/acquisition RDS-SkyCam: 12 Mb/picture (optional lossy compression to 1 Mb/picture)	
Design time life	1.5 Martian Year	

^1^ SA—Shock Absorbers. ^2^ Survival. ^3^ Operational.

**Table 2:** RDS-DP FM performance for the warm case at normal incidence (designed result).

RDS-DP						
TOP Channels	Field of View * (°)	Azimuthal Position (°)	Elevation (°)	Max. Dyn. Range (W/m^2^)	Precision (ppm)	Accuracy (%)
TOP 1—255 ± 5 nm	±15/±12 ± 0.3	162.49	90/89.23	0.05/0.184	1000/78.80	≤10% **/12.0%
TOP 2—295 ± 5 nm	±15/±11.4 ± 0.3	159.88	90/89.27	0.4/1.195	1250/44.1	≤10%/5.5%
TOP 3—250–400 nm	±15/±11. ± 0.3	211.82	90/90.187	60/90.1	1667.7/1.74	≤10%/6.7%
TOP 4—450 ± 40 nm	±15/±11.0 ± 0.3	163.4	90/89.71	80/124	1250/2.44	≤10%/4.5%
TOP 5—650 ± 25 nm	±15/±11.6 ± 0.3	178.14	90/90.166	45/59	2222.2/2.27	≤10%/4.5%
TOP 6—750 ± 10 nm	±15/±10.7 ± 0.3	244.39	90/89.58	15/18	6666.7/2.11	≤10%/4.5%
TOP 7—190–1200 nm	±90/±55 ± 0.3	171.32	90/89.56	600/358 ***	6666.7/1.8	≤10%/5.6%
TOP 8—950 ± 50 nm	±15/±12.1 ± 0.3	185.49	90/90.438	45/64	2222.2/1.8	≤10%/6.5%
LAT Channels	Field of View (°)	Azimuthal Position (°)	Elevation (°)	Max. Dyn. Range (W/m^2^)	Precision (ppm)	Accuracy (%)
LAT 1—750 ± 10 nm	BLIND	0/-	20/-	--	--	--
LAT 2—750 ± 10 nm	±5/±4 ± 0.3	45/45.18	20/21.01	0.12/0.158	1000/25.3	≤10%/6.7
LAT 3—750 ± 10 nm	±5/±4.5 ± 0.3	90/90.9	20/20.14	0.12/0.134	1000/18.9	≤10%/6.7
LAT 4—750 ± 10 nm	±5/±4.5 ± 0.3	135/134.87	20/20.46	0.12/0.144	1000/18.5	≤10%/6.7
LAT 5—750 ± 10 nm	±5/± 4.4 ± 0.3	180/179.95	20/20.38	0.12/0.131	1000/20.2	≤10%/6.7
LAT 6—750 ± 10 nm	±5/±4.2 ± 0.3	225/224.92	20/20.43	0.12/0.157	1000/25.48	≤10%/6.7
LAT 7—750 ± 10 nm	±5/±4.1 ± 0.3	270/269.62	20/20.26	0.12/0.168	1000/20.83	≤10%/6.7
LAT 8—750 ± 10 nm	±5/±4.2 ± 0.3	315/314.94	35/35.91	0.12/0.160	1000/28.31	≤10%/6.7

* Full width at half maximum FWHM. ** Target, not mandatory. *** Parameter below the requirment.

**Table 3:** RDS-SkyCam FM performance.

RDS-SkyCam					
1024 × 1024 CCD	Elevation (°)	Field of View (°)	Dyn. Range (W/m^2^/nm/sr)	Precision (W/m^2^/nm/sr)	Accuracy (%)
	90/89.8 ± 0.1	±62/62.31 ± 0.1 /63.5 ± 0.1 *	≤0.001–0.8 **/3 × 10^−5^−20 ***	0.1/2 × 10^−6^ to 0.1	≤10%/6%

* Diagonal FoV due to the baffle, ** Dynamic range is increased by 10^5^, and precision decreased by 10^5^ with a neutral density coated annulus. *** Using 20 DN in 30 s and 3000 DN in 5 ms.
